# A Computationally Efficient Method for Hybrid EEG-fNIRS BCI Based on the Pearson Correlation

**DOI:** 10.1155/2020/1838140

**Published:** 2020-08-19

**Authors:** Mustafa A. H. Hasan, Muhammad U. Khan, Deepti Mishra

**Affiliations:** ^1^Department of Mechatronics Engineering, Atilim University, Ankara, Turkey; ^2^Department of Computer Science, Norwegian University of Science and Technology (NTNU), Gjøvik, Norway

## Abstract

A hybrid brain computer interface (BCI) system considered here is a combination of electroencephalography (EEG) and functional near-infrared spectroscopy (fNIRS). EEG-fNIRS signals are simultaneously recorded to achieve high motor imagery task classification. This integration helps to achieve better system performance, but at the cost of an increase in system complexity and computational time. In hybrid BCI studies, channel selection is recognized as the key element that directly affects the system's performance. In this paper, we propose a novel channel selection approach using the Pearson product-moment correlation coefficient, where only highly correlated channels are selected from each hemisphere. Then, four different statistical features are extracted, and their different combinations are used for the classification through KNN and Tree classifiers. As far as we know, there is no report available that explored the Pearson product-moment correlation coefficient for hybrid EEG-fNIRS BCI channel selection. The results demonstrate that our hybrid system significantly reduces computational burden while achieving a classification accuracy with high reliability comparable to the existing literature.

## 1. Introduction

In 1924, Hans Berger, a neurologist, recorded human brain signals through EEG for the first time. This actually encouraged other researchers to further investigate the human brain and record its activity using brain computer interface (BCI). This system provides the means for disabled patients to control and communicate with the surroundings, namely, quadcopters, manipulators, and other mechanisms, solely using brain activities. There are different invasive and noninvasive modalities used for BCI applications such as near-infrared spectroscopy (NIRS) [1], functional magnetic resonance imaging (fMRI) [2], and magnetoencephalography (MEG) [3]. In the last decade, most BCI studies have mainly concentrated on electroencephalography (EEG).

EEG, a noninvasive method, registers the electrical activity in the scalp, generated by the brain to provide high temporal resolution with low cost, and portability. Yet, it lacks spatial resolution [4], not to mention poor signal-to-noise ratio, relying on physical or mental tasks, and subject to contamination with various artefacts, such as external electromagnetic waves, e.g., from an electromyogram and an electrooculogram [5]. As a result, the classification accuracy obtained through EEG suffers heavily and often leads to misclassification and false-command generation. Therefore, in order to enhance EEG's performance and make it more reliable, several studies have suggested supporting it with a second modality, such as NIRS [6–9].

Functional near-infrared spectroscopy (fNIRS), another noninvasive method, measures the concentration change of oxyhemoglobin (HbO) and deoxyhemoglobin (HbR) by picking two distinct near-infrared (NIR) wavelengths (600 nm to 1000 nm). fNIRS offers subsecond temporal resolution and spatial resolution in 1 cm^2^ [10] and has shown its potential in localizing task activations in the same way as fMRI. However, fNIRS has some serious concerns that constrains the overall performance. Contrary to EEG, NIRS is considered to have strong immunity against electrical noises and motion artefacts; however, it suffers from long delay in the hemodynamic response. The response time in order to generate the execution command for NIRS is approximately nine times that of EEG [7]. Over the past few years, researchers have been striving to increase the information transfer rates (ITR) and to overcome the limitations of unimodal systems, leading to multimodal ones, formally known as a hybrid BCI.

In [11], the authors made the first attempt to investigate a hybrid EEG-fNIRS-based BCI system, and to integrate the features of EEG and fNIRS, they designed a metaclassifier for the hybrid BCI. Since then, many other studies have addressed the benefits of the complementary properties of integrated EEG-fNIRS [6, 7, 9, 12]. This combination enhanced the signal strength, improved the classification accuracy, and overcame most of the limitations of single modalities [6, 11, 13]. The main challenge in a hybrid BCI is to reduce system complexity, improve response time, and maintain high classification accuracy [1, 14, 15]. Though, the multimodal BCI system has enhanced the classification accuracy, methodological constraints still exist, such as heavy reliance on the principle component analysis (PCA) and common spatial pattern (CSP) [16, 17], empirical wavelet transform (EWT) [18], and multivariate empirical wavelet transform (MEWT) [19] to reduce the dimensionality of the original dataset. Another motivation behind the integration of EEG-fNIRS is to retain and highlight the favourable properties of each unimodal system [20].

In hybrid BCI studies, three major steps define the overall performance of the system: channel selection, feature extraction, and classification. This study focuses on the identification and selection of most optimal channels with the intention to reduce the computational cost while maintaining high classification accuracy. In previous studies [1, 7], all available channels from both hemispheres were considered for feature extraction and classification. This not only increased system complexity but also computational cost.

To confront these issues, various methods have been proposed in the near past. It is worth mentioning that most of the literature has merely focused on crude analysis to select the fewer channels, manually [21–23]. This approach may work in some cases, but the generalization of such methods is limited by self-analysis as well as by the excessive time required in order to analyse each individual channel. In contrast, some researchers endeavoured for the sophisticated approaches as how to determine the optimal channels. In [9], the authors proposed to select a singular channel of EEG-fNIRS from both hemispheres using a general linear model (GLM); however, the obtained average classification accuracy was moderate. Also, the performance of GLM might be subjected to artefacts that infect the data, such as motion artefacts, low frequency inclines, and serial correlations [24]. Common spatial patterns (CSPs) have also been utilized to determine the most effective channels [25]. Based upon the average energy of each channel, the ones with the highest energy are selected; whereas, those with low energy are assumed to carry noise and, hence, set aside.

In this paper, we present a novel approach for a hybrid EEG-fNIRS BCI channel selection using the Pearson product-moment correlation coefficient (PPMCC). In recent years, researchers have tried to explore the benefits of correlation in BCI for the window selection [26], channel selection [27], and classification [28]. The determination of the correlation coefficient is a statistical approach that allows us to quantify the strength of association. The calculation yields the linear association between two channels which is then used to select the most dominant ones. Based upon the correlation coefficient, a rank matrix is developed. The highest ranked channels are classified as active channels or true motor imagery signals, whereas the lowest ranked channels are regarded as nonrepresentative channels of motor imagery signals. Those with lower correlation coefficients can also be interpreted as artefacts, noise, or other signals that are not correlated with motor imagery signals. To the best knowledge of the authors, PPMCC has never been utilized for channel selection in a BCI paradigm before. Therefore, this study is expected to introduce a completely new perspective for channel selection in hybrid BCI systems. Through results, it is demonstrated that the proposed approach can play its role effectively, when used in conjunction with two different classifiers, to attain reduced computational time and high classification accuracy. The following sections present the signal processing, feature extraction, and classification approaches.  In Results, the outcomes are shown with focus on the performance.  Discussion and Conclusion conclude the work by summarizing the findings and proposed future work.

## 2. Materials and Methods

### 2.1. Data Source and Data Processing

**Figure 1:**
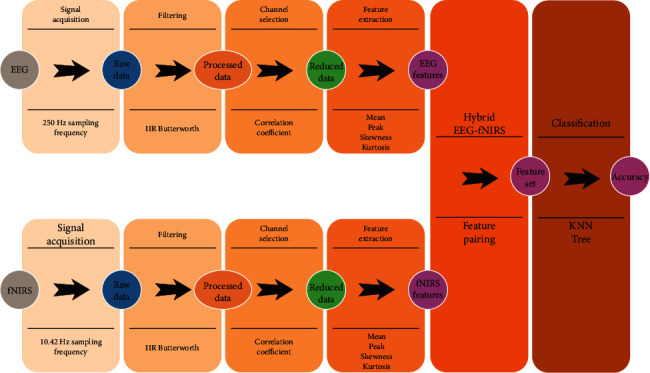
Hybrid BCI system.

The dataset for this study is taken from an online repository, provided by [6]. It contains raw data of simultaneously recorded EEG-fNIRS motor tasks of the left arm, the left hand, the right arm, and the right hand. For motor imagery tasks, fifteen healthy subjects took part in the experiment, which lasted an hour. During the experiment, the subjects were instructed to perform four movements—right–left-hand gripping, right–left-arm raising—by following visual instructions displayed on a laptop screen placed 1 m away from where they were sitting. For each subject, the trials started with a rest task of 6 seconds, followed by another 6 seconds of movements according to the screen instructions. The raw EEG signals were obtained at 250 Hz through twenty-one channels, all of which were baseline corrected by subtracting the mean value of channels and then filtered at 1-25 Hz by a 4th-order IIR Butterworth filter [9]. The fNIRS signals were acquired at a sampling frequency of 10.42 Hz for two wavelengths—760 nm and 850 nm—equipped with twelve sources and twelve electrodes on thirty-four channels. The sources and detectors of the fNIRS are at maximum 3.4 cm apart from each other, in order to ascertain high-quality signals. The raw data is later decomposed into oxyhemoglobin and deoxyhemoglobin concentration changes (HbO and HbR, respectively) through the Modified Beer-Lambert law [29]. In this study, we used HbO, referred to as fNIRS from here on. The fNIRS data is filtered by a 4th-order band pass IIR Butterworth filter (between 0.01 and 0.2 Hz) to remove artefacts [6, 9]. Initialization trial prior to the motor tasks was segmented out. The filtered EEG and fNIRS data are then normalized by subtracting the mean and dividing by the standard deviation.  Figure 1 shows the hybrid BCI system that highlights all the major steps involved.

### 2.2. Channel Selection for Hybrid BCI

In hybrid BCI studies, channel selection is considered as the key element that directly affects the system's performance. The Pearson correlation coefficient has been applied by a number of researchers [27, 30, 31] in order to solve practical problems in medical research. Although it has given some promising results, it has never been tested for hybrid BCI systems before. Hence, we propose a novel approach to ensure optimal performance by involving only the most optimized channels using the Pearson product-moment correlation coefficient. The approach is based upon statistical analysis that allows to quantify the strength of a linear association between two channels, denoted by *ρ*, having a value between [−1, 1]. The basic idea is to associate the data of two channels through the best fit line. The Pearson correlation coefficient, *ρ*, is an indicator of the placement of these data points in reference to the line of best fit. A higher positive value indicates a stronger association, whereas a more negative value is an indicator of a stronger negative association. The third possibility is absolutely no association between the variables, i.e., *ρ* = 0.

**Figure 2:**
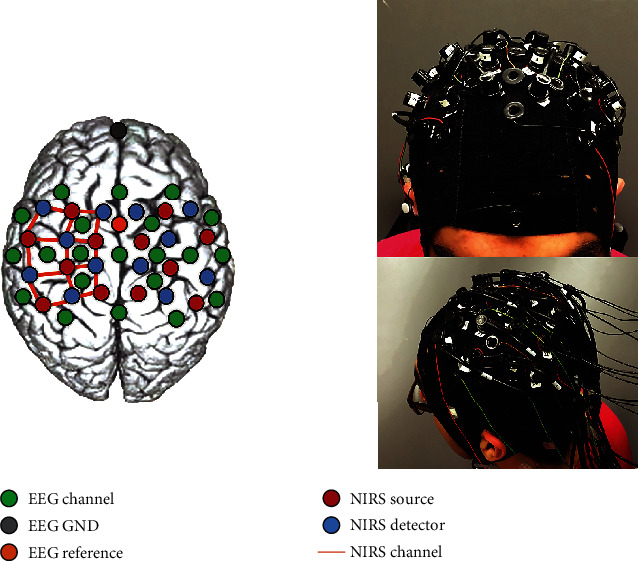
EEG-fNIRS channel placement [6].

EEG-fNIRS channels are distributed into two groups based upon their placement in the right and left hemispheres, as shown in Figure 2. The correlation coefficient between the two intervals in each group is a measure of how close a linear relationship these two intervals possess. Given a pair of two channels [*i*, *j*], the correlation coefficient is defined as follows:

(1) $$\begin{array}{c} \rho_{i,j}=\frac{E\left[\left(i-\mu_{i}\right)\left(j-\mu_{j}\right)\right]}{\sigma_{i}\sigma_{j}}, \end{array}$$

where *μ* represents the mean value, *σ* denotes the standard deviation, and *E* represents the expectation operator.

The reference guideline for interpreting the strength of association based upon the correlation coefficient is given in Table 1. For the purpose of selecting the most representative channels (see Algorithm 1) with the most meaningful performance, and to eliminate signals with unnecessary information and noise, highly correlated EEG-fNIRS channels from each hemisphere are chosen based upon their ranking. To reduce dimensions and improve the parity of the two systems, the EEG-fNIRS dataset is downsized through a 0.096 sec averaged moving window.

### 2.3. Feature Extraction

Once the channels are selected, the next task is to prepare a feature set for classification. Four different statistical features are extracted using spatial averaging of the selected channels for both EEG and fNIRS features.

#### 2.3.1. Signal Mean (*M*)

For channel *X* acquired through EEG-fNIRS, the signal is contained in *x*_1_ through *x*_*N*_, then the mean value for the discrete signal is evaluated as follows:

(2) $$\begin{array}{c} M=\frac{1}{N}\overset{N}{\underset{i=1}{\sum }}X_{i}, \end{array}$$

where *N* is the total number of observations.

#### 2.3.2. Signal Skewness (SK)

The skewness of a channel *X* is the third standardized moment, represented as follows:

(3) $$\begin{array}{c} \text{SK}\left(X\right)=E\left[{\left(\frac{X-\mu }{\sigma }\right)}^{3}\right]. \end{array}$$

The distribution of *X* is said to be positively skewed (SK > 0), negatively skewed (SK < 0), or unskewed (SK = 0).

#### 2.3.3. Signal Kurtosis (KR)

The kurtosis of *X* is the fourth standardized moment, defined as follows:

(4) $$\begin{array}{c} \text{KR}\left(X\right)=E\left[{\left(\frac{X-\mu }{\sigma }\right)}^{4}\right]. \end{array}$$

Kurtosis is an indicator of the peak of the distribution. For positive it is peaked more, and for negative it is peaked less than the normal distribution.

#### 2.3.4. Signal Peak (*P*)

The peak *X*_*p*_ of a channel *X* is defined as *X*_*i*_ ≤ *X*_*p*_, 1 ≤ *i* ≤ *N*.

Once the feature set is defined, the next process is to normalize the feature set of both EEG-fNIRS between 0 and 1 using

(5) $$\begin{array}{c} X_{\text{new}}=\frac{X_{i}-\min\left(X_{i}\right)}{\max\left(X_{i}\right)-\min\left(X_{i}\right)}. \end{array}$$

From here on, it is assumed that all the features are normalized, hence new feature vectors are obtained as *M*_new_, SK_new_, KR_new_, and *P*_new_. But, in order to avoid the ambiguity and for the sake of easiness, the normalized features are represented as *M*, SK, KR, and *P*.

### 2.4. Classification

Prior to classification, three different sets of features are constructed: the EEG-only set, the fNIRS-only set, and the hybrid EEG-fNIRS set. Two different classifiers, *K*-nearest neighbor (KNN) and Decision Tree classifier, are used to perform classifications among five different classes: four motor tasks and one rest task. Both classifiers belong to the supervised learning and are widely used due to their simplicity and ease of implementation. A 10-fold crossvalidation paradigm is implemented to split each feature set into ten subsets, where nine subsets are used to train the classifiers, and the remaining one subset is used to test the classification accuracy. Through the results, it is observed that both classifiers have performed well in order to attain high classification accuracy.

## 3. Results

The goal of this study is to perform a comparison among three different sets of features: EEG-only, fNIRS-only, and hybrid EEG-fNIRS for the selected channels based on a correlation coefficient. To achieve this, we performed two-trial classifications of the four motor execution tasks versus rest. A classification accuracy of 100% refers to perfectly separated motor tasks, whereas 50% signifies poor performance. The movements of the subjects are recorded through twenty-one EEG and thirty-four fNIRS channels in total.

**Figure 3:**
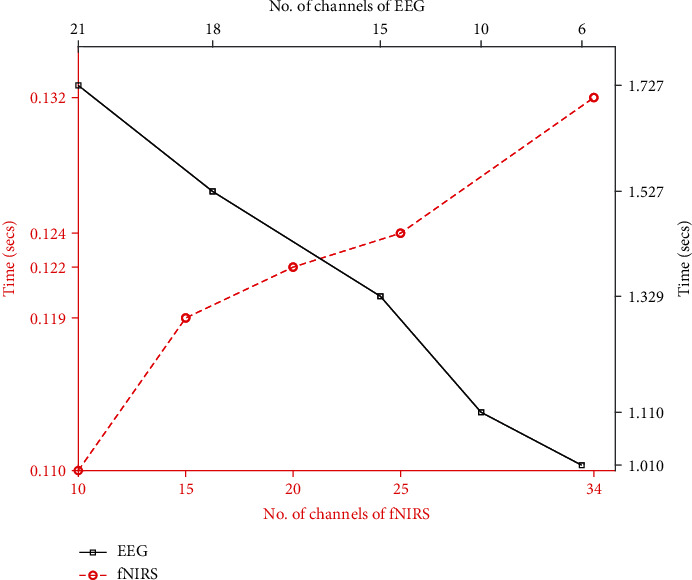
Performance comparison of EEG and fNIRS.

To analyse the reduction in the computational time, the temporal distance between the filtration step and feature extraction is recorded as an evaluation time. Comparison is made for 10 sec of the sampled data obtained through EEG and fNIRS.  Figure 3 shows the computational time required in order to process a hundred and twenty-five-hundred samples of fNIRS and EEG, respectively, against a varying number of channels. It can be observed that the relation between the number of channels and computational time is almost linear. Thus, by utilizing a lesser number of channels, time reduction is expected. Through the proposed approach, only highly correlated channels are obtained and processed. For EEG, only six channels are selected out of twenty-one, and for fNIRS, only ten channels are selected out of thirty-four. The processing time is reduced by more than 40% for EEG and around 20% for fNIRS. In this study, it has been observed that for the experiments considered, if we further try to reduce the number of channels, the accuracy will start to deteriorate with not much improvement in time. In the later part of this section, it is revealed that only those channels that are the carrier of less significant information compared to the rest are dropped. This helps to get rid of the outliers and noise variation that may have been introduced in some channels at the time of data acquisition.

**Figure 4:**
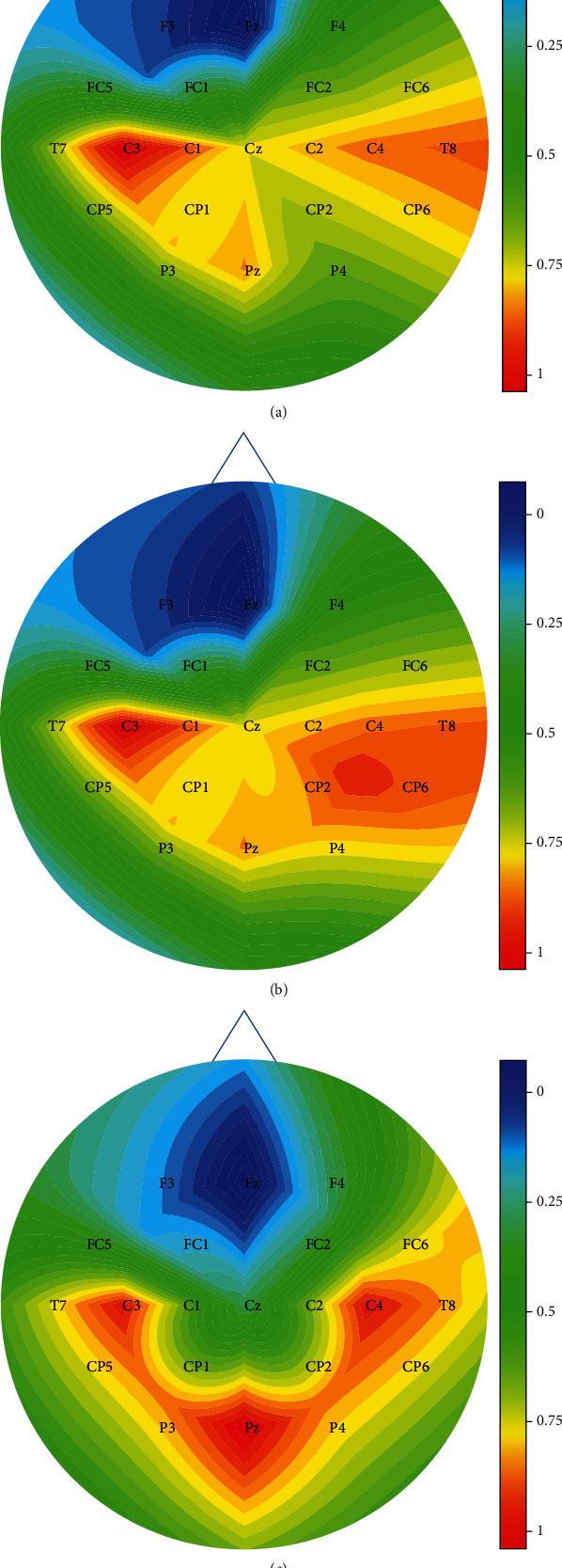
Probability of channels being selected based upon correlation coefficients of (a) subject 1, (b) subject 4, and (c) subject 15.

With the help of a novel correlation coefficient-based method, only those channels that contain the most relevant motor imagery information are picked. The probability of the channels being selected based upon the correlation results obtained for subjects 1, 4, and 15 is depicted in Figure 4. The highest correlation coefficients are obtained in the motor cortex region in the right and left hemispheres. According to Figure 4, this is true because the movements considered are of the left arm, left hand, right arm, right hand, and rest. The highest correlation coefficient helps to separate the optimized channels from the rest. Once the channels are selected, the next task is to train the classifiers on the given dataset from different sources in order to obtain accurate results.

### 3.1. EEG

**Figure 5:**
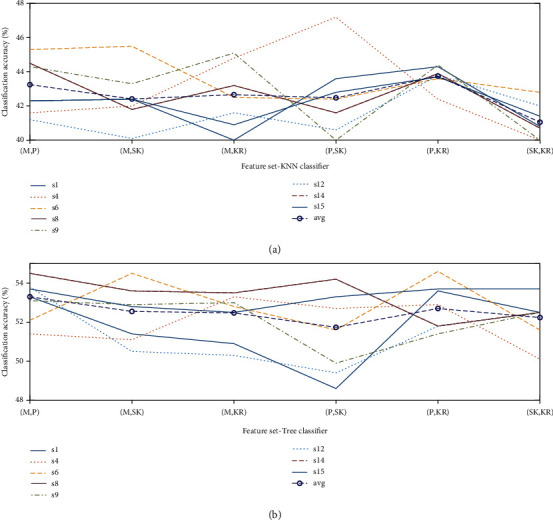
Classification accuracies obtained using EEG when evaluated for eight subjects: (a) KNN classifier and (b) Tree classifier.

**Figure 6:**
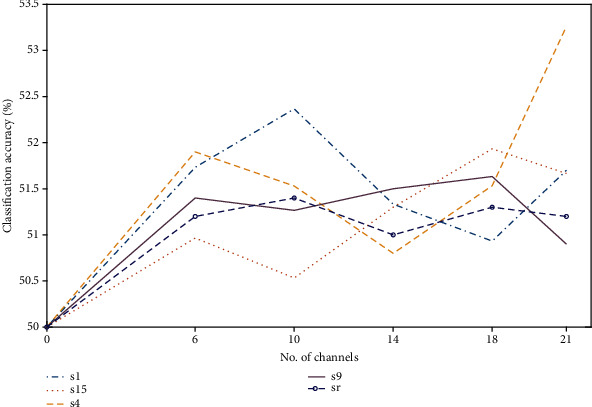
Classification accuracies obtained using EEG when evaluated for five subjects against a varying number of channels.

The average classification accuracies for five different classes (four movements, one rest) obtained using KNN and Tree classifiers are shown in Table 2. Mean (*M*), peak (*P*), kurtosis (KR), and skewness (SK) are defined as statistical features. The reduced number of channels from eight subjects are processed to generate a feature set that contains all one-to-one possible combinations of the features. The Tree classifier has produced acceptable results when evaluated against KNN for the given feature set. The average classification accuracy obtained through KNN is well below the defined acceptable threshold of 50%. This phenomenon has occurred, according to our observation, as some of the subjects were not presumably fully engaged in the task and lost interest at some stage during the process. This caused an overall drop in the accuracy, thus the motivation to eliminate the channels that directly affects the accuracy. In order to observe the performance of eight individual subjects, the classification results obtained through both classifiers using the optimized channels are plotted in Figure 5. In accordance with the results obtained in Table 2, it is reaffirmed that the KNN classifier has not been able to produce any satisfactory results, whereas the Tree classifier hardly satisfies the defined acceptable threshold. The average classification accuracies of the EEG-only feature set achieved through the Butterworth filter are KNN = 42.72% and Tree = 52.49%. The classification accuracies obtained through the Tree classifier for the selected subjects against a varying number of channels are plotted in Figure 6. The feature set is based upon the data obtained from five selected subjects including randomly selected (sr). It is observed that an increase in the number of channels has not produced any significant effect on the accuracy but certainly increased the computational cost.

### 3.2. fNIRS

**Figure 7:**
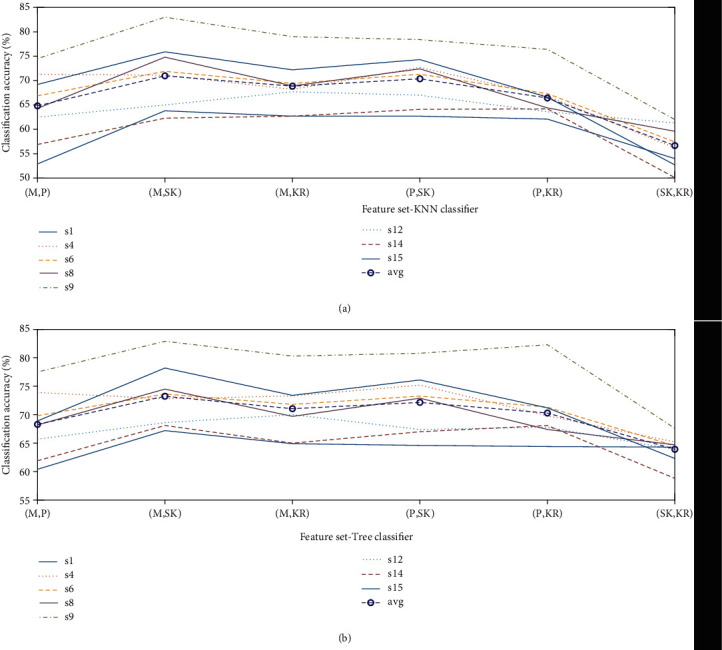
Classification accuracies obtained using fNIRS when evaluated for eight subjects: (a) KNN classifier and (b) Tree classifier.

Table 2 shows the KNN and Tree classification results—using the most optimized channels from eight subjects—for the fNIRS-only dataset. In this study, it is revealed that, for fNIRS, the combination of peak and skewness has produced the highest accuracy for both classifiers. The accuracy obtained through fNIRS is much higher as compared to EEG. To highlight the performance variation among subjects, classification accuracies based upon eight individuals—along with their average—are depicted in Figure 7. The fNIRS-only feature set filtered by the Butterworth filter achieved an average accuracy of KNN = 66.34% and Tree = 69.82%.

**Figure 8:**
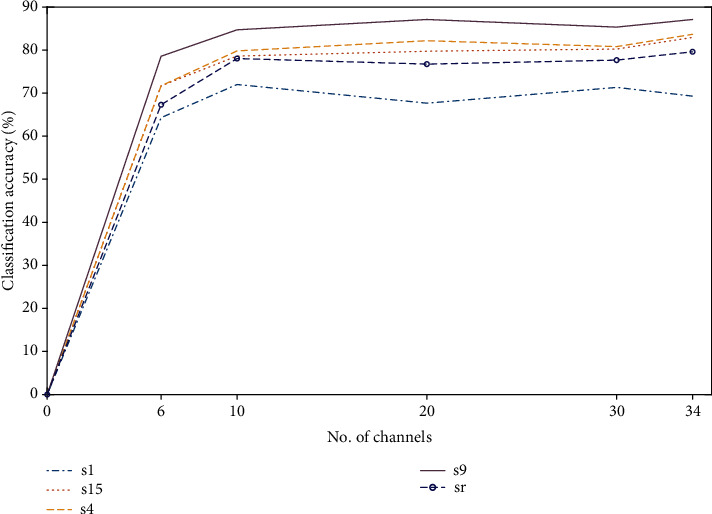
Classification accuracies obtained using fNIRS when evaluated for five subjects against a varying number of channels.

It is observed that some subjects have really outperformed others; e.g., subject nine can be regarded as the healthiest among the sample set, whose accuracy is well above the average accuracy for fNIRS. Thus, it can be concluded that for the overall high accuracy, every subject is supposed to perform at their best.  Figure 8 shows the average classification accuracies using the Tree classifier for five selected subjects including randomly selected (sr), when evaluated against a varying number of channels. Again, subject nine has been able to perform the best as compared to the others. The increase in the number of channels did not play a big role in improving the accuracy; in fact, if we select less than 30% of the total number of channels, then the accuracy drops significantly.

### 3.3. Hybrid EEG-fNIRS

For the hybrid EEG-fNIRS, the EEG feature set contains six feature vectors that are combined with the fNIRS feature set, which also contain the same number of feature vectors, in order to generate a total of thirty-six feature vectors.  Table 3 shows the performance in terms of the average classification accuracy for eight subjects obtained through KNN and Tree classifiers. No absolute judgement can be made regarding the most desired feature set; however, [M, SK] has performed well as compared to the rest at multiple instances. The highest and the lowest average classification accuracies of 78.2% and 66.7% are obtained through the Tree classifier, whereas the highest and lowest average classification accuracies obtained through the KNN classifier are 75.28% and 60.76%.

**Figure 9:**
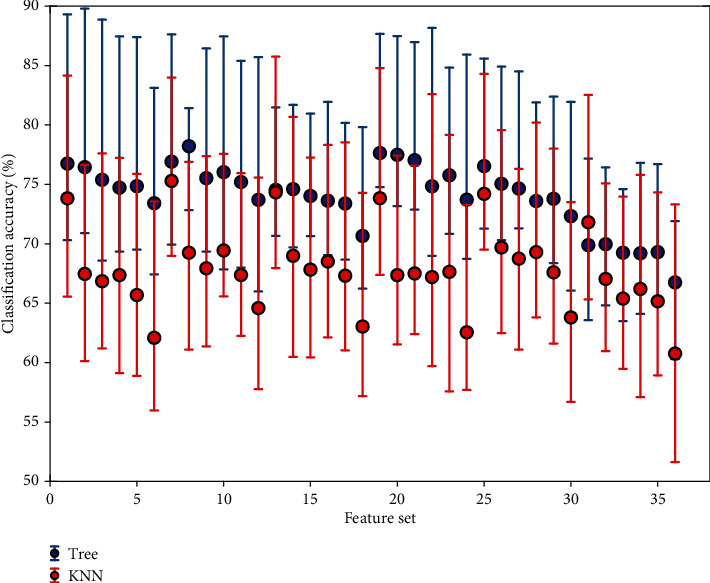
Classification accuracies obtained using hybrid EEG-fNIRS when evaluated for eight subjects.

Figure 9 shows the classification accuracies for eight subjects obtained through the KNN and Tree classifiers against different features; moreover, the average accuracies are also depicted through filled circles. It is observed that against some feature vectors, the Tree classifier has been able to get as high as 90% classification accuracy. Overall, the Tree classifier has proved to be more effective when compared against KNN for the hybrid EEG-fNIRS BCI system.

To evaluate the performance of the proposed approach, it is compared against [6], who provided the actual dataset. The classification accuracy is obtained through a linear discriminant analysis (LDA) classifier for the rest vs. the right-hand movement. For true analysis, the type of classifier and the movement is kept the same as [6]. For EEG-only, fNIRS-only, and hybrid EEG-fNIRS, our approach achieves the classification accuracy of 61.6%, 98%, and 98.6%, respectively. The classification results obtained by [6] for EEG-only, fNIRS-only, and hybrid EEG-fNIRS are 85.4%, 92.4%, and 94.2%, respectively. It can be observed that at two out of three instances, the proposed approach performed better against Buccino et al. [6], who considered all channels.

## 4. Discussion

The hybrid BCI using EEG-fNIRS has proved itself capable of improving classification accuracy as compared to a single modality [11]. In this paper, we attempted to improve the classification accuracy of motor tasks as well as to reduce the computational cost. The results demonstrate that the EEG-fNIRS combination based upon the selection of the most optimized channels has performed better compared to the unimodal approaches. It is widely accepted that the design and application of a BCI system is strongly influenced by the selection of channels, their number, and their placement [9].

To generate execution commands based upon motor imagery tasks, previous studies have recommended picking channels from the C3 and C4 areas [6, 11]. Yet, this approach cannot be generalized for all due to variations from subject to subject, as identical channels may exist in different brain regions. Although some researchers investigated the efficiency of different channel selection approaches [7, 17, 33], little effort has been made in the selection of the most optimized channels. In this study, we use a set of channels from each hemisphere with high similarities based on the correlation coefficient to ensure that the most effective channels are chosen for feature extraction and classification. The obtained results show that not only did the classification accuracy increase but the computational complexity also reduced. As Table 3 summarizes the classification accuracy of the merged features of EEG-fNIRS, it is noticeable that although few channels are used for the classification, the accuracy tended to be decent as compared to previous studies.

The classification performance of a full channel set does not necessarily yield an optimal performance as witnessed in Figures 6 and 8. Thus, it is desired that only those sets of channels that actually contain a significant amount of information must be considered. Through the selection of the most optimized channels, the processing time has improved by 40% for EEG and around 20% for fNIRS. The channel selection approach based on the ranking of the correlation coefficient can identify the optimal channel combination and enhance the classification performance. The classification accuracy of the system can further be increased by selecting optimal features for the system. Signal mean, peak, skewness, and kurtosis are used as the feature set for the classification. It has been shown that the use of these sets of features achieved high accuracy in fNIRS classification, yet the accuracy in EEG is low due to the dimensionality issue and, as mentioned by [6], the experimental design and procedure strongly affect the performance.

## 5. Conclusions

In this study, a hybrid EEG-fNIRS configuration is proposed for motor task classification. The primary goal of this work is to reduce the computational cost by not compromising the classification accuracy. In order to realize such a system, for the first time we propose to utilize a correlation coefficient for the selection of the most optimized channels. To validate the effectiveness of the proposed approach, eight different subjects are considered and multiple trials are performed. As evident from the results, our hybrid system significantly reduces the computational burden while achieving the classification accuracy with high reliability comparable to the existing literature.

Despite this accomplishment, we observed two major limitations that hold back the overall accuracy of the hybrid BCI system. The motor imagery task data was collected with an EEG visual feedback system. Through the visual information displayed on the screen, the subjects were instructed to perform certain movements. The motor classification accuracy of 52.49 ± 4% is achieved through EEG only, which is not even comparable to the accuracy of 72.42 ± 3% obtained through fNIRS using the Tree classifier. This phenomenon has also been experienced by some researchers when dealing with EEG [34]. This is perhaps because the subjects' involved in the experiments were exposed to the motor imagery tasks for the first time. Therefore, in order to improve the results in motor imagery tasks, it has been suggested in [35] that all the subjects be trained for 1-4 hours with visual feedback informing the user whether his/her imagery strategy is correctly classified. Secondly, as EEG suffers from low spatial resolution, it directly affects the overall performance of the hybrid system. The use of high-resolution EEG can possibly overcome this problem. As to future work, we focus on the validation of the results from the practical perspective [36–38] and the investigation of the performance of the entire system using online data. Furthermore, different feature sets will be explored in our studies to further improve the classification of the system.

## Figures and Tables

**Algorithm 1:**
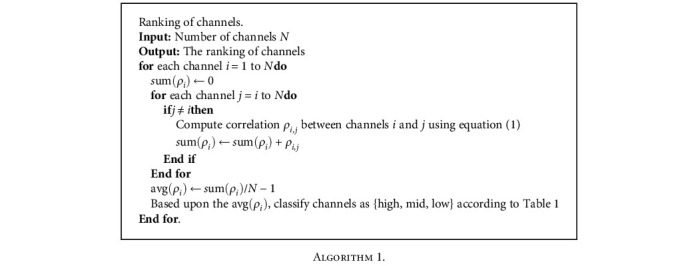


**Table 1:** Correlation coefficient [32].

Strength of association	Coefficient, *ρ*	
	Positive	Negative
Small	0.1 to 0.3	-0.1 to -0.3
Medium	0.3 to 0.5	-0.3 to -0.5
Large	0.5 to 1.0	-0.5 to -1.0

**Table 2:** The classification accuracies obtained through KNN and Tree classifiers for EEG-only and fNIRS-only feature sets.

Feature set	KNN		Tree	
	EEG	fNIRS	EEG	fNIRS
*M*, *P*	43.25	64.81	**53.30**	68.29
*M*, SK	42.41	**70.97**	52.55	**73.24**
*M*, KR	42.66	68.85	52.47	71.05
*P*, SK	42.48	70.36	51.73	72.16
*P*, KR	**43.75**	66.43	52.70	70.29
SK, KR	41.05	56.67	52.23	63.91

**Table 3:** The average classification accuracies of hybrid EEG-fNIRS for eight subjects as calculated through KNN and Tree classifiers.

Feature set fNIRS	Feature set EEG					
	*M*, *P*	*M*, SK	*M*, KR	*P*, SK	*P*, KR	SK, KR
*M*, *P*	(73.82, 76.75)	(67.46, 76.45)	(66.85, 75.38)	(67.36, 74.72)	(65.68, 74.85)	(62.08, 73.4)
*M*, SK	(**75.28**, 76.91)	(69.25, **78.2**)	(67.93, 75.5)	(69.43, 76.02)	(67.37, 75.2)	(64.58, 73.7)
*M*, KR	(74.32, 74.53)	(68.98, 74.6)	(67.82, 74.02)	(68.51, 73.62)	(67.31, 73.38)	(63.03, 70.6)
*P*, SK	(73.83, 77.63)	(67.36, 77.4)	(67.5, 77.03)	(67.2, 74.83)	(67.63, 75.76)	(62.55, 73.7)
*P*, KR	(74.2, 76.53)	(69.68, 75)	(68.75, 74.65)	(69.3, 73.6)	(67.6, 73.78)	(63.8, 72.32)
SK, KR	(71.81, 69.88)	(67.03, 69.9)	(65.38, 69.25)	(66.2, 69.2)	(62.16, 69.3)	(60.76, 66.7)

## Data Availability

The raw data used to support the findings of this study are deposited in EEG-fNIRS hybrid SMR BCI data S1-S8 and EEG-fNIRS hybrid SMR BCI data S9-S15.
